# Increasing prevalence of anticholinergic medication use in older people in England over 20 years: cognitive function and ageing study I and II

**DOI:** 10.1186/s12877-020-01657-x

**Published:** 2020-07-31

**Authors:** Carlota M. Grossi, Kathryn Richardson, George M. Savva, Chris Fox, Antony Arthur, Yoon K. Loke, Nicholas Steel, Carol Brayne, Fiona E. Matthews, Louise Robinson, Phyo K. Myint, Ian D. Maidment

**Affiliations:** 1 University of East Anglia, Norwich, UK; 2 University of East Anglia, Quadram Institute Bioscience, Norwich Research Park, Norwich, UK; 3 University of Cambridge, Cambridge, UK; 4 University of Newcastle, Newcastle, UK; 5 University of Aberdeen, Aberdeen, UK; 6 Aston University, Birmingham, B4 7ET UK

**Keywords:** Cognitive impairment, Anticholinergic burden

## Abstract

**Background:**

Anticholinergic medication use is linked with increased cognitive decline, dementia, falls and mortality, and their use should be limited in older people. Here we estimate the prevalence of anticholinergic use in England’s older population in 1991 and 2011, and describe changes in use by participant’s age, sex, cognition and disability.

**Methods:**

We compared data from participants aged 65+ years from the Cognitive Function and Ageing Studies (CFAS I and II), collected during 1990–1993 (*N* = 7635) and 2008–2011 (*N* = 7762). We estimated the prevalence of potent anticholinergic use (Anticholinergic Cognitive Burden [ACB] score = 3) and average anticholinergic burden (sum of ACB scores), using inverse probability weights standardised to the 2011 UK population. These were stratified by age, sex, Mini-Mental State Examination score, and activities of daily living (ADL) or instrumental ADL (IADL) disability.

**Results:**

Prevalence of potent anticholinergic use increased from 5.7% (95% Confidence Interval [CI] 5.2–6.3%) of the older population in 1990–93 to 9.9% (9.3–10.7%) in 2008–11, adjusted odds ratio of 1.90 (95% CI 1.67–2.16). People with clinically significant cognitive impairment (MMSE [Mini Mental State Examination] 21 or less) were the heaviest users of potent anticholinergics in CFAS II (16.5% [95% CI 12.0–22.3%]). Large increases in the prevalence of the use medication with ‘any’ anticholinergic activity were seen in older people with clinically significant cognitive impairment (53.3% in CFAS I to 71.5% in CFAS II).

**Conclusions:**

Use of potent anticholinergic medications nearly doubled in England’s older population over 20 years with some of the greatest increases amongst those particularly vulnerable to anticholinergic side-effects.

## Background

Globally, the population is ageing; in the UK, the proportion of people aged 65 years or over is projected to increase from 18% in 2017 to 21% by 2027 [1]. Multi-morbidity increases with ageing. This increase has been accompanied by a dramatic rise in polypharmacy with the proportion of older people taking five or more medication rising four-fold from 12 to 49% over 20 years [2]. The concerns about polypharmacy include interactions, burden on patients, side effects, and cost. Many older people frequently receive medicines with anticholinergic properties for diverse conditions, such as depression, bladder problems, Parkinson’s disease and chronic obstructive pulmonary disease [3–6].

Anticholinergic activity can cause cognitive decline, falls, constipation and daytime drowsiness in older people [7, 8], and worsen cognition and activities of daily living in people living with schizophrenia [9]. Greater cumulative use of anticholinergics has been associated with an increased risk of dementia [10–12], and mortality [7]. Given these possible associations with long term outcomes as well as the known immediate adverse anticholinergic effects, it is widely accepted that these medicines should be avoided in older people where possible [13]. Nevertheless, anticholinergics remain commonly prescribed.

Estimates of prevalence of anticholinergic use vary depending on the population, year and definition of anticholinergic medications. Previous estimates of the prevalence of any anticholinergic use in older adults have varied from 37 to 63% [8, 14], and of ‘potent’ anticholinergic use from 4 to 10% [8, 14, 15]. However, less is known about the impact of anticholinergic effects among groups most vulnerable to their side-effects such as older people with clinically significant cognitive impairment including those living with dementia and the very old, because these groups are commonly excluded from clinical trials [16, 17].

Medications with anticholinergic activity are most commonly available only by prescription, but are also obtainable over-the-counter. Hence pharmacy dispensing or prescription databases may underestimate the true prevalence of anticholinergic medication use in the population. Prospective longitudinal studies, which aim to ascertain participants’ over the counter (OTC) and prescription medication use, may offer the best opportunities to understand changing patterns in their use. These prospective longitudinal studies also allow the disaggregation of older populations by health status and so allow the medication use patterns to be described by physical and cognitive frailty.

The overall aim of the study was to estimate the prevalence of anticholinergic use in England’s older population in 1991 and 2011, and describe changes in use by participant’s age, sex, cognition and disability.

## Methods

This study compared the prevalence of anticholinergic medication use in the older population of England using baseline data from two prospective longitudinal studies conducting using identical methods in 1990/1993 and 2008/2011. The authors assert that all procedures contributing to this work comply with the ethical standards of the relevant national and institutional committees on human experimentation and with the Helsinki Declaration of 1975, as revised in 2008. All procedures involving human subjects were approved by local and multi-centre ethical committees (CFAS I: REC99/5/22, 05/MRE05/37; CFAS II: 07/MRE05/48).

Data were obtained from the first waves of the Cognitive Function and Ageing Studies: CFAS I and CFAS II. The CFAS studies are population-based longitudinal studies of ageing, where participants aged 65 and older were randomly selected from the Family Health Service Authority lists from specific areas of England and Wales, and include community-based participants and those in long-term care.

Potential participants were initially contacted via a letter from their general practice. If the potential participant provided written informed consent, this was followed by a visit from a trained interviewer (often from a health-related background) from a team of interviewers. The interview was conducted in the participants’ place of residence using a structured, computer assisted interview with direct data entry.

For potential participants considered to lack mental capacity, as per the Mental Capacity Act in the UK, a request to a key informant, usually a close family member, was made for an interview. The interviews were then performed with the assistance of a proxy (often a close family member).

Participants were interviewed between December 1990 and July 1993 (CFAS I), and between November 2008 and October 2011 (CFAS II).

Data from three centres from CFAS I of Cambridgeshire (rural), Newcastle (urban) and Nottingham (urban) were chosen to match the centres in CFAS II. Sampling was stratified by age group (65–74 vs ≥75 years). CFAS I and CFAS II had very similar designs and assessment methods and so medication use prevalence estimates can be directly compared [18]. The response rate was 80% in CFAS I and 56% in CFAS II [18]. Inverse probability weights are available for both studies to ensure estimates reflect the age and sex structure of their respective populations. CFAS I and II assessments include questions about socio-demographic characteristics, residence (long-term care or community-dwelling), medications, health, activities of daily living (basic and instrumental), and tests of cognitive function [18]. For data access and study information visit www.cfas.ac.uk.

### Medication exposure

Medication usage, both prescribed and over the counter, was obtained by self-report. At interview, participants were asked ‘Are you currently taking any medicines, tablets or injections of any kind, either you buy yourself or are prescribed by your doctor?’ Where possible packaging was checked, with proxies supplying medication information if required. All prescribed and over the counter medications were recorded using NHS Read codes [2]. Read codes are a computerised comprehensive coded thesaurus used within the NHS. Prescription and OTC medication reported was scored 0 to 3 on the Anticholinergic Burden Scale (ACB). Medication with in-vitro activity, but no clinically relevant effect are scored 1. Medication with clinically relevant effects are scored 2 or 3 if associated with delirium. Other medications are scored 0. For the full list visit:

https://www.uea.ac.uk/documents/3306616/10940915/Anticholinergics/088bb9e6-3ee2-4b75-b8ce-b2d59dc538c2

Details of the scale development are reported elsewhere [4, 5]. For medications available in the UK, but not rated on the ACB scale, we applied the same approach used and thus scored (i) all thiazide diuretics, loop diuretics and antihistamines as 1, (ii) all tricyclic antidepressants as 3, and (iii) all creams, eye and ear drops as 0. CFAS did not record how medications were obtained, and some common medications with potential anticholinergic properties such as chlorphenamine, ranitidine, cimetidine, and codeine products could be prescribed or purchased OTC. We defined any anticholinergic use as the use of any medications scoring 1, 2 or 3 on the ACB scale, potent anticholinergic use as any scoring 3 on the ACB scale, and the anticholinergic burden as the sum of ACB scores for all medications taken.

### Population subgroups

We estimated anticholinergic use in groups defined by sex, age (grouped in 5-year age bands), cognitive function (Mini-Mental State Examination [MMSE] ≤21, MMSE 22–25, and MMSE 26–30 points) [19], and disability [measured by impairments in modified Townsend activities of daily living (ADL) and instrumental activities of daily living (IADL)] [20].

### Statistical analysis

We estimated the prevalence of any anticholinergic medication use, potent anticholinergic use and the average anticholinergic burden, using inverse probability weights that accounted for non-response [18]. To compare cohorts, estimates were standardised to the 2011 UK age and sex distribution, using 5-year age bands, to account for changes in population structure. Prevalences were also estimated in the pre-defined population subgroups. Participants with missing disability or MMSE data were excluded from those comparisons.

We used logistic regression to estimate odds ratios (OR) for ‘potent’ and ‘any’ anticholinergic use in CFAS II compared to CFAS I. We used negative binomial regression to estimate the rate ratio comparing the anticholinergic burden between the two cohorts, as anticholinergic burden was an over-dispersed discrete variable. The differences between cohorts were adjusted for age, sex and centre and weighted for non-response. We also tested for interaction effects between subgroups and CFAS cohort to identify different trends over time among the different groups of the older population.

Finally, the prevalence of the potent anticholinergic medication by urological, antispasmodic, antipsychotic, antidepressant, anxiolytic, parkinsonian and antihistamine classes was estimated in CFAS I and II.

## Results

### Population characteristics

CFAS I and II included data from 7635 and 7762 participants, respectively. Table 1 summarises the characteristics of CFAS I and II participants. Although the mean and Standard Deviation (SD) age were similar, 75.3 (7.1) for CFAS I vs 75.7 (7.3) for CFAS II, there was a greater proportion aged over 85 years in CFAS II. Participants of CFAS II were also slightly more likely to be men and have more IADL disability than in CFAS I.

**Table 1:** Baseline participant characteristics in CFAS I and CFAS II

Demographic characteristics	CFAS I (***N*** = 7,635)	CFAS II (***N*** = 7,762)
**Sex**		
Male	3045 (39.9)	3534 (45.5)
Female	4590 (60.1)	4228 (54.5)
**Age**		
64–69	1981 (25.9)	1939 (25.0)
70–74	1776 (23.3)	1873 (24.1)
75–79	1725 (22.6)	1624 (20.9)
80–84	1308 (17.1)	1278 (16.5)
85–89	615 (8.1)	737 (9.5)
90+	230 (3.0)	311 (4.0)
**Centre**		
Cambridgeshire	2601 (34.1)	2558 (33.0)
Newcastle	2522 (33.0)	2582 (33.3)
Nottingham	2512 (32.9)	2622 (33.8)
**MMSE**^**1**^		
Median (IQR)	27 (24, 28)	28 (26, 29)
**Disability**		
None	5236 (68.6)	4975 (64.1)
IADL disability	1048 (13.7)	1495 (19.3)
ADL-IADL disability	1267 (16.6)	981 (12.6)
**Residence**		
Community-dwelling	7245 (94.9)	7565 (97.5)
Long term care	242 (3.2)	197 (2.5)

Cell entries denote n (%) unless otherwise specified

1139 and 255 participants had missing MMSE data in CFAS I and CFAS II

*Abbreviations*: *CFAS* Cognitive Function and Ageing Studies, *CI* confidence interval, *MMSE* Mini-Mental State Examination, *ADL* activities of daily living, *IADL* instrumental activities of daily living, *SD* standard deviation, *IQR* Interquartile range

### Potent anticholinergic use

The overall prevalence of potent anticholinergic use among the over 65s increased from 5.7% (95% CI 5.2–6.3%) to 9.9% (95% CI 9.3–10.7%) between CFAS I and II. After adjusting for demographic differences, the odds ratio for this increase was 1.90 (95% CI 1.67–2.16) (Table 2).

**Table 2:** Prevalence of any and potent anticholinergic use in CFAS I and CFAS II, by age, sex, cognition and disability

	Any anticholinergic use				Potent anticholinergic use			
Population	Prevalence % (95% CI)		Adjusted OR for CFAS II vs CFAS I^**a**^		Prevalence % (95% CI)		Adjusted OR for CFAS II vs CFAS I^**a**^	
	CFAS I	CFAS II	OR (95% CI)	p^**b**^	CFAS I	CFAS II	OR (95% CI)	p^**b**^
**Overall**	49.6 (48.4, 50.7)	64.3 (63.2, 65.4)	1.25 (1.17, 1.34)	< 0.001	5.7 (5.2, 6.3)	9.9 (9.3, 10.7)	1.90 (1.67, 2.16)	< 0.001
**By sex**								
Male	46.7 (44.9, 48.6)	61.3 (59.6, 62.9)	1.00	0.06	3.6 (3.0, 4.4)	6.4 (5.6,7.3)	1.00	0.59
Female	51.3 (49.8, 52.8)	66.7 (65.2, 68.2)	1.14 (0.99, 1.30)		7.0 (6.3, 7.8)	12.8 (11.7,13.9)	1.08 (0.82, 1.43)	
**By age, years**								
64–69	44.4 (42.2,46.6)	53.5 (51.2,55.7)	1.00	< 0.001	5.2 (4.3,6.3)	8.0 (6.8,9.5)	1.00	0.49
70–74	48.2 (45.9,50.6)	62.9 (60.7,65.1)	1.33 (1.11, 1.60)		5.9 (4.9,7.1)	9.9 (8.6,11.4)	1.11 (0.76, 1.60)	
75–79	52.8 (50.4,55.2)	68.8 (66.4,71.0)	1.41 (1.17, 1.71)		6.0 (4.9,7.2)	11.3 (9.7,13.0)	1.28 (0.88, 1.86)	
80–84	54.3 (51.5,57.0)	73.0 (70.4,75.5)	1.48 (1.20, 1.82)		6.8 (5.5,8.3)	11.0 (9.3,12.9)	1.09 (0.73, 1.62)	
85–89	53.8 (49.6,58.0)	72.1 (68.5,75.5)	1.58 (1.21, 2.07)		5.3 (3.7,7.5)	11.8 (9.5,14.6)	1.59 (0.95, 2.69)	
90+	50.7 (43.5,57.8)	75.5 (68.9,81.1)	2.10 (1.36, 3.22)		4.1 (2.0,8.0)	9.0 (5.9,13.6)	1.54 (0.63, 3.75)	
**By cognition**								
MMSE ≤21	53.3 (49.0, 57.6)	71.5 (65.0, 77.1)	1.00	0.02	11.2 (8.6,14.4)	16.5 (12.0, 22.3)	1.00	0.83
MMSE 22–25	52.7 (50.2, 55.1)	69.9 (67.1, 72.6)	0.94 (0.70, 1.25)		6.7 (5.6,8.1)	13.4 (11.4,15.6)	1.10 (0.71, 1.70)	
MMSE 26–30	48.6 (47.0, 50.2)	62.8 (61.5, 64.1)	0.76 (0.59, 0.99)		4.5 (3.9,5.2)	8.4 (7.7,9.2)	1.00 (0.68, 1.49)	
**By disability**								
No impairment	42.4 (40.8,44.0)	56.9 (55.4,58.5)	1.00	0.27	3.8 (3.3, 4.5)	6.3 (5.6, 7.1)	1.00	0.05
IADL impairment	67.1 (64.0,70.0)	80.4 (78.1,82.5)	1.13 (0.93, 1.36)		6.8 (5.4, 8.7)	15.8 (13.8, 18.0)	1.46 (1.04, 2.04)	
ADL impairment	71.1 (67.7,74.3)	85.4 (82.3,88.1)	1.15 (0.92, 1.43)		15.8 (13.1,18.9)	20.8 (17.6, 24.5)	0.95 (0.70, 1.29)	

Weighted for nonresponse and standardised by the UK 2011 age population, missing cases excluded

*Abbreviations*: *CFAS* Cognitive Function and Ageing Studies, *CI* confidence interval, *MMSE* Mini-Mental State Examination, *ADL* activities of daily living, *IADL* instrumental activities of daily living

^a^Adjusted for age, sex and centre

^b^Global test for the interaction between the covariate and difference in prevalence between CFAS I and CFAS II

In CFAS II, 12.8% of women used a potent anticholinergic compared to 7.0% of men. This is approaching twice the rate in CFAS I for both sexes. Potent anticholinergic use was not strongly related to age; but the heaviest users in CFAS II were those with clinically significant cognitive impairment (16.5% [95% CI 12.0–22.3%] of those with an MMSE of 21 or less) and more disability, with 20.8% (95% CI 17.6–24.5%) of the most disabled using a potent anticholinergic compared to 6.3% (95% CI 5.6–7.1%) of those with no disability. The greatest rate of increase between cohorts was seen among those with IADL disability (from 6.8% [95% CI 5.4–8.7%] in CFAS I to 15.8% [95% CI 13.8–18.0%] in CFAS II, *p*-value for interaction = 0.05).

The increases in potent anticholinergic use were driven by an increased use of anticholinergic urologicals and antidepressants (Table 3). Use of potent anticholinergic urologicals and antidepressants increased from 0.3% (95% CI 0.2–0.4%) to 2.8% (95% CI 2.4–3.2%) and 4.0% (95% CI 3.6–4.5%) to 5.9% (95% CI 5.4–6.5%) between CFAS I and CFAS II, respectively. The most common anticholinergic urologicals used in CFAS II were oxybutynin (35% of anticholinergic urological drugs), tolterodine (31%) and solifenacin (17%), and the most common anticholinergic antidepressant reported in CFAS II was amitriptyline (69% of anticholinergic antidepressant drugs).

**Table 3:** Prevalence of potent anticholinergic use in CFAS I and CFAS II, by drug class

Potent anticholinergic class	1991 CFAS I	2011 CFAS II
Urological	0.3 (0.2, 0.4)	2.8 (2.4, 3.2)
Antispasmodic	0.5 (0.3, 0.6)	0.3 (0.2, 0.5)
Antipsychotic	1.0 (0.8, 1.2)	0.9 (0.7, 1.1)
Antidepressant	4.0 (3.6, 4.5)	5.9 (5.4, 6.5)
Anxiolytic	N/A	0.2 (0.1, 0.3)
Parkinsonian	0.2 (0.2, 0.4)	0.1 (0.0, 0.2)
Antihistamine	0.2 (0.1, 0.3)	0.5 (0.3, 0.7)

Cell entries denote % prevalence (95% confidence intervals)

*Abbreviations*: *CFAS* Cognitive Function and Ageing Studies

### Any anticholinergic use

The prevalence of medication use with ‘any’ anticholinergic activity increased from 49.6% (95% CI 48.4–50.7%) to 64.3% (95% CI 63.2–65.4%) between CFAS I and CFAS II (Table 2, adjusted OR of 1.25; 95% CI 1.17–1.34). The greatest increases in use across the 20 years was observed for older participants (from 50.7% [95% CI 43.5–57.8%] in CFAS I to 75.5% [95% CI 68.9–81.1%] in CFAS II for those aged 90 years or more, *p*-value for interaction < 0.001), and in those with clinically significant cognitive impairment (from 53.3% [95% CI 49.0–57.6%] in CFAS I to 71.5% [95% CI 65.0–77.1%] in CFAS II for those with an MMSE score of 21 or lower, *p*-value for interaction = 0.02).

### Anticholinergic burden

The average total anticholinergic burden increased from 0.99 (95% CI 0.96–1.03) in 1991 to 1.11 (95% CI 1.08–1.15) in 2011, adjusted ratio of 1.12 (95% CI 1.07–1.17) (Table 4).

**Table 4:** Average anticholinergic burden in CFAS I and CFAS II, by age, sex, cognition and disability

Population	Mean ACB sum (95% CI)		Adjusted rate ratio for CFAS II vs CFAS I^**a**^	
	CFAS I	CFAS II	Rate ratio (95% CI)	p^**b**^
**Overall**	0.99 (0.96, 1.03)	1.11 (1.08, 1.15)	1.12 (1.07, 1.17)	< 0.001
**By sex**				
Male	0.91 (0.86, 0.95)	0.96 (0.92–1.01)	1.00	0.01
Female	1.05 (1.01, 1.09)	1.23 (1.18–1.28)	1.12 (1.03, 1.23)	
**By age, years**				
64–69	0.87 (0.81, 0.92)	0.81 (0.75–0.87)	1.00	< 0.001
70–74	0.97 (0.91, 1.04)	1.10 (1.03–1.66)	1.21 (1.05, 1.38)	
75–79	1.09 (1.02, 1.15)	1.26 (1.18–1.34)	1.24 (1.09, 1.42)	
80–84	1.12 (1.04, 1.20)	1.28 (1.19–1.36)	1.22 (1.06, 1.41)	
85–89	1.07 (0.95, 1.18)	1.37 (1.25–1.49)	1.39 (1.17, 1.66)	
90+	0.97 (0.77, 1.17)	1.36 (1.17–1.54)	1.53 (1.17, 1.99)	
**By cognition**				
MMSE ≤21	1.29 (1.13, 1.46)	1.32 (1.13, 1.52)	1.00	0.03
MMSE 22–25	1.09 (1.02, 1.16)	1.38 (1.28, 1.48)	1.11 (0.94, 1.31)	
MMSE 26–30	0.93 (0.89, 0.97)	1.04 (1.01, 1.09)	0.97 (0.83, 1.13)	
**By disability**				
No impairment	0.76 (0.72, 0.79)	0.85 (0.81, 0.89)	1.00	0.91
IADL impairment	1.51 (1.40, 1.61)	1.64 (1.54, 1.74)	1.02 (0.92, 1.13)	
ADL impairment	1.85 (1.71, 1.99)	1.88 (1.74, 2.03)	1.02 (0.91, 1.14)	

Prevalence (95% confidence interval) displayed, weighted for nonresponse and standardised by the UK 2011 age population, with missing cases excluded

*Abbreviations*: *ACB* Anticholinergic Cognitive Burden scale, *CFAS* Cognitive Function and Ageing Studies, *MMSE* Mini-Mental State Examination, *ADL* activities of daily living, *IADL* instrumental activities of daily living

^a^Adjusted for age, sex and centre

^b^Global test for the interaction between the covariate and ratio of total anticholinergic burden between CFAS I and CFAS II

Women and older participants had the greatest total burden score, and had experienced the greatest increases since CFAS I. For example the mean ACB score increased from 1.05 (95% CI 1.01–1.09) in CFAS I to 1.23 (95% CI 1.18–1.28) in CFAS II for women (p-value for interaction = 0.01), and from 0.97 (95% CI 0.77–1.17) in CFAS I to 1.36 (95% CI 1.17–1.54) in CFAS II for those aged 90 years or more (p-value for interaction< 0.001).

## Discussion

The prevalence of potent anticholinergic use in the older population in England nearly doubled between 1990/93 and 2008/11. After adjustment for demographic variables, we found that participants in the later study (CFAS II) were 1.9 times more likely to be on potent anticholinergics as compared to participants in the earlier study (CFAS I). This was mainly due to increases in the availability and use of anticholinergic urologicals (common drugs were oxybutynin, solifenacin and tolterodine) and antidepressants (the most common being amitriptyline). More than one in five of those with an impairment in activities of daily living, and one in six of those with MMSE less than 21, indicating clinically significant cognitive impairment including dementia, reported use of a potent anticholinergic medication in CFAS II, both significantly higher than in CFAS I [21]. This is despite guidance suggesting cautious use of these drugs. Those with IADL disability had the greatest disproportionate increases in potent anticholinergic use. Women and older participants also had disproportionately greater increases in total anticholinergic burden between study periods.

A number of studies have described changes in the rates of anticholinergic prescribing [16, 17]. A study in Scotland examined changes in the numbers of prescriptions of anticholinergic medications, from 1995 to 2010, and found a statistically significant but modest increase in the number of older people prescribed any anticholinergic (20.7% vs 23.7%, *p* < 0.001) [16]. A repeated cross-sectional analysis of office-based outpatient visits for older people in the USA found that the prevalence of high-risk anticholinergic prescriptions was stable from 2006 to 2015; it increased from 6.1% in 2006–07 to 6.8% in 2008–09 and decreased to 4.7% by 2014–15 [17]. However, previous studies have not been able to include over-the-counter medication use nor describe use in vulnerable patient groups; the US study also only included prescriptions issued by the physician at the sampled visit.

We observed an increase in anticholinergic urological use between 1991 and 2011, partly because many of the commonly used urologicals were only introduced in the 1990s, or later. Other studies have also reported increases in the prescribing of anticholinergic urologicals [16, 22, 23]. A 23% increased number of new users of anticholinergics for overactive bladder was reported in a UK study (from 12,598 in 2004 to 15,441 in 2012) [23]. A significant increase in the proportion of women presenting to physicians with urinary incontinence then prescribed bladder anticholinergics was also reported in the US (16.7% in 1999 to 35.0% in 2009, *p* = 0.006) [22].

Use of anticholinergic antidepressants also increased between CFAS I and II; confirming other studies [24]. In addition to anticholinergic effects, antidepressants are associated with hyponatraemia [25, 26]. Even mild hyponatraemia induced by antidepressants may worsen cognition and cause falls compounding apparent anticholinergic effects [25]. Depression is also an early sign of dementia and therefore older people with depression may be particularly vulnerable to cognitive anticholinergic effects [27].

Anticholinergics can have a significant impact on morbidity in older people particularly those living with any form of clinically significant cognitive impairment including dementia [7, 12]. Anticholinergics can worsen dementia, cause numerous anticholinergic effects, both centrally and peripherally, and may be associated with an excess mortality [7, 12]. Equally importantly, anticholinergics could also worsen the quality of life of the older person and any informal (family) carer [28].

The large increase in the use of potent anticholinergics among people with clinically significant cognitive impairment and physical disabilities is particularly concerning. There is increasing evidence, from recent research, that such usage is associated with an increased risk of dementia [10, 11, 12]. Furthermore, anticholinergic cognitive effects are likely to have more severe consequences, such as medication errors, in people with less cognitive reserve for example dementia or traumatic brain injury [29]. Medication management itself is an instrumental activity of daily living with high demands on memory and executive function [30], and so anticholinergic induced cognitive impairment may increase the risk of both non-adherence to medication, and medication errors [30, 29]. This in turn will increase dependency on informal carers worsening the burden on informal carers [31].

### Strengths and weaknesses

Frail older people with multi-morbidities including those with dementia are frequently excluded from controlled trials, and so effectiveness of anticholinergics is rarely directly assessed, and observational studies are vital for monitoring risks. Strengths of this study include the population-based sampling in CFAS from the same geographic areas 20-years apart and to ascertain key patient characteristics, cognition and disability associated with medication use. Although most anticholinergics are prescribed, a further strength of our study was the ability to more accurately capture the full range of anticholinergic use, by including OTC medications.

This appropriateness of prescribing was not assessed as part of the CFAS study. The increase in use of anticholinergics might reflect improvements in diagnosis and better access to treatment for conditions such as incontinence, depression and pain. Such conditions can be very debilitating, and for clinicians and patients the key issue is balancing the risks versus the benefits.

Our study has some limitations. The accuracy of the self-reported medication use and the duration of treatment is unknown. Although, to increase the accuracy of the reporting, interviewers requested, where possible, to see the medication packages (and repeat prescription scripts) to enter correct drug names, we cannot be sure if the participants were adherent to the medication. The data used is from 1990 to 1993 and 2008 to 2011 and therefore we recommend that the study is repeated with more recent data to examine whether the trends continue. Studies examining UK trends in anticholinergic medication use post 2011 are rare. Increased prescribing of anticholinergics for overactive bladder has been reported until 2012 for adults [23]. Warnings against antipsychotic use in dementia has decreased prescribing to these patients [32], but we lack information on the general older population. Antidepressant prescribing has been increasing from 2013 to 18, but detail has not been provided by anticholinergic antidepressants or for older people specifically [33].

We used the ACB scale to identify anticholinergic medications, however this is one of 18 different scales that all vary in their content and how they are derived and how anticholinergic activity is quantified [34]. However, the scales closely agree on which medications they classify as potently anticholinergic. The response rate was lower in CFAS II, and it is not clear whether this would under-estimate or over-estimate medication use in this cohort. We used inverse probability weights to correct age and sex distributions for non-response, and conducted analyses stratified by levels of cognitive function and disability, and so our findings are unlikely to be biased by differential non-response between cohorts [18]. Our study is descriptive and we did not have sufficient comorbidity data to sufficiently examine why older people in the various subgroups had increased anticholinergic use, but increased diagnoses of conditions for which anticholinergics are indicated for is likely a factor.

### Future research

Further research is needed to monitor anticholinergic use within vulnerable populations, particularly older people living with clinically significant cognitive impairment including dementia, in the UK since 2011 and in other countries. We also need a clearer understanding of the relative risk versus benefit of anticholinergics and in whom the risk is greatest, and the effectiveness of interventions to reduce the harm associated with anticholinergics. Interventions to limit the use of inappropriate anticholinergics require development and testing; a realist approach, which focuses on the key importance of context and mechanism offers a promising avenue for such intervention development [35–37].

## Conclusions

In summary the use of potent anticholinergics nearly doubled in the older population in England over an appropriate 20 year period (from 1990/93 to 2008/11), largely due to rising use of antidepressants and urologicals. The use of anticholinergics is highest among the most vulnerable groups including people living with clinically significant cognitive impairment. This raises concerns as anticholinergic medications are associated with a range of side-effects including cognitive decline.

## Data Availability

Data can be shared through application. For further information please refer to the application forms on the website http://www.cfas.ac.uk/cfas-i/data/#cfasi-data-request

## Abbreviations

ACB: Anticholinergic Cognitive Burden
ADL: Activities of daily living
CFAS: Cognitive Function and Ageing Studies
CI: Confidence Interval
IADL: Instrumental activities of daily living
MMSE: Mini Mental State Examination
NHS: National Health Service
OR: Odds ratios
OTC: Over the counter
SD: Standard Deviation
